# Preoperative non-invasive visual localization of synchronous multiple lung cancers using three-dimensional computed tomography lung reconstruction

**DOI:** 10.1186/s13019-021-01666-w

**Published:** 2021-09-26

**Authors:** Guofei Zhang, Duo Xu, Zipu Yu, Lian Wang, Haihua Gu, Ying Chai, Gang Shen

**Affiliations:** 1 Department of Thoracic Surgery, The Second Affiliated Hospital, Zhejiang University School of Medicine, 88 Jiefang Road, Hangzhou, 310009 China; 2 Department of Radiology, The Second Affiliated Hospital, Zhejiang University School of Medicine, 88 Jiefang Road, Hangzhou, 310009 China

**Keywords:** Non-invasive visual localisation, Synchronous multiple primary lung cancers, Three-dimensional lung reconstruction, Video-assisted thoracoscopic surgery

## Abstract

**Background:**

Synchronous multiple primary lung cancers are becoming more common with increasing use of computed tomography for screening. Intraoperative localization and resection of ill-defined pulmonary ground-glass opacities during thoracoscopic resection is challenging. This study aimed to determine the clinical feasibility of non-invasive visual localization of these nodules by three-dimensional computed tomography lung reconstruction before sublobar resection.

**Methods:**

Forty-four patients with synchronous multiple primary lung cancers underwent thoracoscopic pulmonary resection at our institution between June 2017 and August 2019. Preadmission computed tomography images were downloaded and reconstructed into a three-dimensional model. Small nodules (< 15 mm) were localized non-invasively by three-dimensional computed tomography lung reconstruction before surgery. Patient demographics, nodule characteristics, procedural details, pathological data, and outcomes were obtained from the medical records.

**Results:**

One hundred and twenty-one pulmonary nodules from the 44 patients were scheduled for video-assisted thoracic surgery; 54 (44.6%) were pure ground-glass opacities and 57 (47.1%) were mixed ground-glass opacities. One hundred and seventeen nodules were localized preoperatively. The mean nodule diameter was 7.67 ± 3.87 mm. The mean distance from the nodule to the pleura was 14.84 ± 14.43 mm. All nodules were removed successfully by wedge resection (27 patients), lobectomy (26 patients), or segmentectomy (25 patients). Most lesions (85.1%) were malignant. Paraffin pathology revealed 12 cases of atypical adenomatous hyperplasia (9.92%), 13 of adenocarcinoma in situ (10.74%), 16 of minimally invasive adenocarcinoma (13.22%), and 73 of invasive adenocarcinoma (60.33%).

**Conclusions:**

Three-dimensional computed tomography lung reconstruction is a feasible and alternative method of visual localization for small lung nodules before sublobar resection in some suitable patients.

## Introduction

Synchronous multiple primary lung cancers (SMPLC) are presumed to be uncommon. However, their true incidence (0.2–20%) is increasing because of the widespread use of early detection tools, such as high-resolution computed tomography (CT) [1]. When the main lesion is removed, the remaining small lesions are always removed as far as possible. Sublobar resections, including wedge resection and segmentectomy, under video-assisted thoracoscopic surgery (VATS) are widely used, especially for semi-solid or ground-glass nodules (GGN) [2]. However, non-solid pulmonary nodules judged to be too small or located too deeply beneath the pleural surface to be seen or palpated during thoracoscopy must be localised preoperatively[3, 4]. Current preoperative techniques that facilitate localisation of ground-glass opacities (GGO) include microcoil or hookwire placement; however, these methods have logistic limitations, are associated with safety concerns, increase the complexity of the procedure, and expose patients to further radiation [5–8]. Moreover, CT-guided percutaneous localisation methods have several “blind areas”, including the region in the vicinity of the mediastinum, areas neighbouring the interlobar fissure, and scapulae-shadowed areas [9]. Furthermore, the economic expenditure and risks are also increased in patients with SMPLC.

Preoperative three-dimensional computed tomography bronchography and angiography (3D-CTBA) can demonstrate the segmental structures, reveal anatomic variations, and improve the accuracy of surgery. The location of the nodule can also be identified intuitively and accurately on 3D-reconstructed images according to the relationship between the nodule and the reconstructed segmental border, which is useful for accurate targeted removal of the segment containing the nodule [10]. However, when using this method, it is easy to misjudge the location of the nodule during wedge resection.

Three-dimensional computed tomography lung reconstruction (3D-CTLR) can accurately determine the relative position of a nodule in the lobe. For a segmentectomy or wedge resection, it allows non-invasive visual localisation of nodules. At our institution, we routinely used 3D-CTBA for segmentectomy until 2017; since then, we use 3D-CTLR for localisation when performing uniportal VATS for SMPLC. Here we report our experience of 44 procedures using 3D-CTLR to localise small lung nodules before uniportal VATS resection. We also aimed to determine the clinical feasibility of this technique for precise localisation of small nodules before sublobar resection.

## Methods

Forty-four patients (13 male, 31 female; mean age, 55.6 years) were retrospectively identified as having undergone preoperative localisation of SMPLC by 3D-CTLR before sublobar resection between June 1, 2017 and August 31, 2019 at the Second Affiliated Hospital Zhejiang University School of Medicine. The main indication for surgery was enlargement of the main nodule on follow-up CT images. Clinical parameters, including patient age and sex, size, number and site of nodules, surgical procedure performed (wedge resection, segmentectomy, or lobectomy), operating time, duration of postoperative hospital stay, and in-hospital morbidity and mortality, were collected from the patients’ charts. The histopathology of the resected nodules was classified according to the criteria set by the World Health Organization in 2015.

The nodules were further classified according to the results of percentage quantification of GGO as pure GGN, partially solid GGN with GGO ≥ 50%, partially solid GGN with GGO < 50%, and pure solid nodules. The operating surgeon determined the eligibility of patients with lung nodules that required non-invasive visual localisation before wedge resection by VATS when the maximum diameter of the target lung nodule was < 15 mm.

### Three-dimensional CT lung and nodule reconstruction

**Fig. 1:**
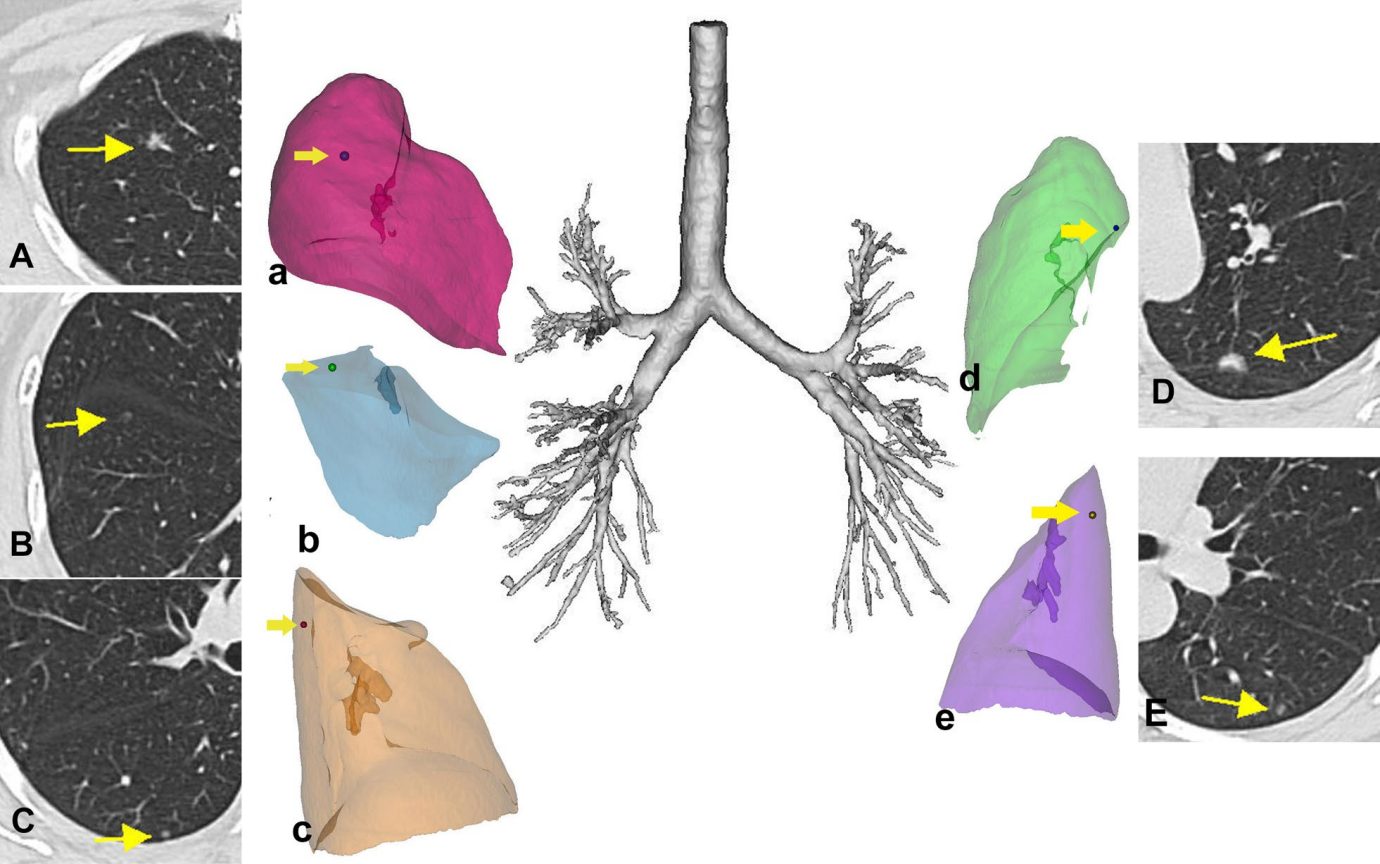
The reconstructed lobes and nodules for patient 1. **A**–**E** Computed tomography images showing the primary lesions as ground-glass opacities (yellow arrow) in the five lobes. (a–e) Three-dimensional computed tomography lung reconstruction displaying the nodules (yellow arrow) and the reconstructed lungs in the real relative position. Postoperative pathologic findings confirmed adenocarcinoma in situ (**E**), minimally invasive adenocarcinoma (**B**, **C**) and invasive adenocarcinoma (**A**, **D**)

High-resolution CT scans with a slice thickness of 1.5 mm are acquired routinely in patients with small lung nodules at our institution. We use commercially available medical image processing software (MIMICS [Materialise Interactive Medical Image Control System], version 20.0, Materialise, Belgium) to process the CT data obtained and create an accurate 3D model for the patient. The MIMICS can create masks and 3D models of the left and right lung as well as separate 3D models for each of the lung lobes. The MIMICS software can also reconstruct the pulmonary nodules and display the reconstructed lobes and nodules in their real relative positions (Fig. 1). The 3D-CTLR is completed at least 1 day before the scheduled surgery.

### Non-invasive visual localisation procedure

When the 3D reconstruction of the lobes and nodules is completed, we can see the position of the nodules relative to the lobes. The method used for accurate localisation of these nodules is in accordance with the principle of individualisation and is described in the following sections.

#### Superficial pulmonary nodules

##### Certain specific parts of the lobes

**Fig. 2:**
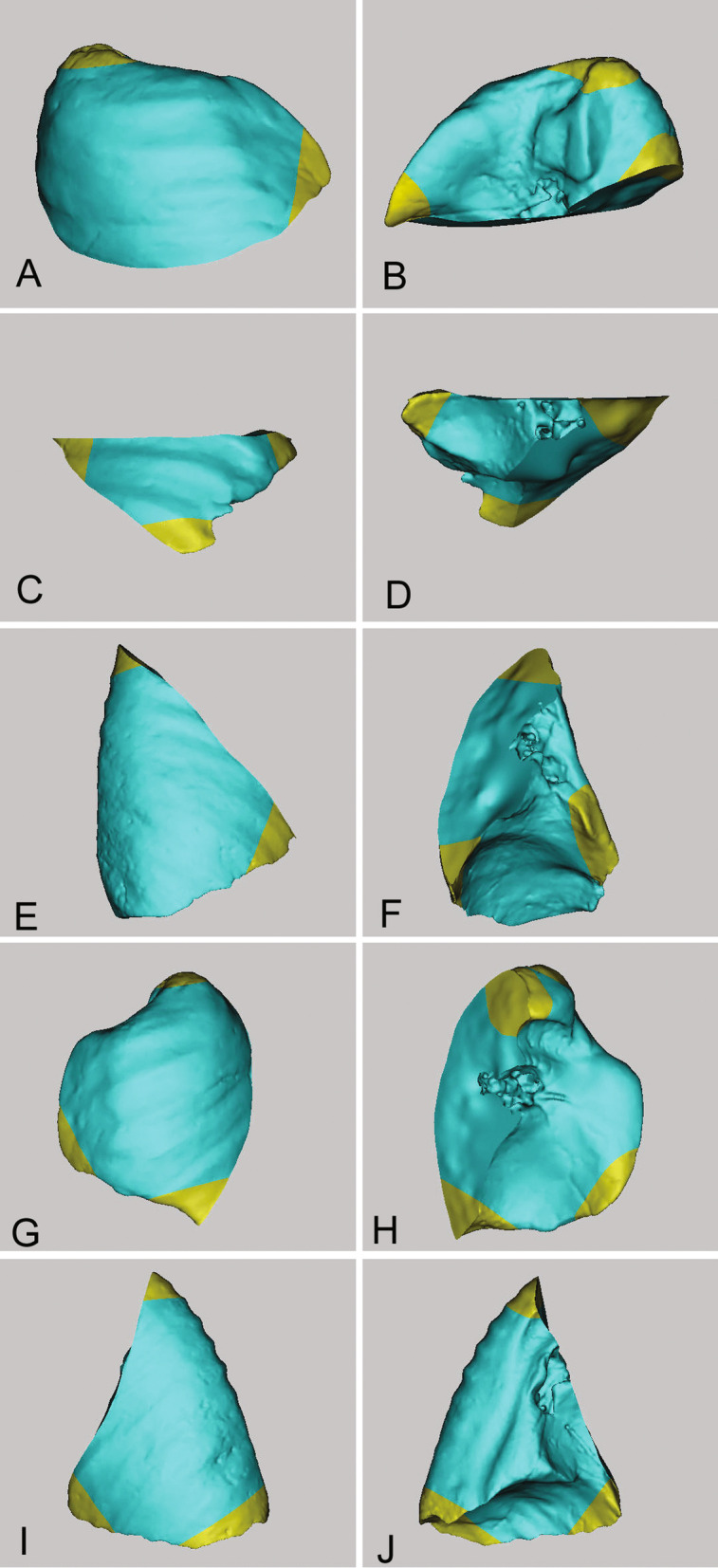
Several specific parts of the lobes (yellow parts). The nodules in these parts of the lobes could be directly removed by wedge resection. (**A**, **C**, **E**, **G**, **I**) Lateral aspect of the five lobes. (**B**, **D**, **F**, **H**, **J**) Medial aspect of the five lobes

For superficial pulmonary nodules, wedge resections are schematised. Nodules in certain specific parts of the lobes, such as the tip of the upper lobe, the upper portion of the dorsal segment of the lower lobe, and the attachment area of multiple plane junctions of each lobe can be removed directly by wedge resection (Fig. 2).

##### Fixed anatomic landmarks

**Fig. 3:**
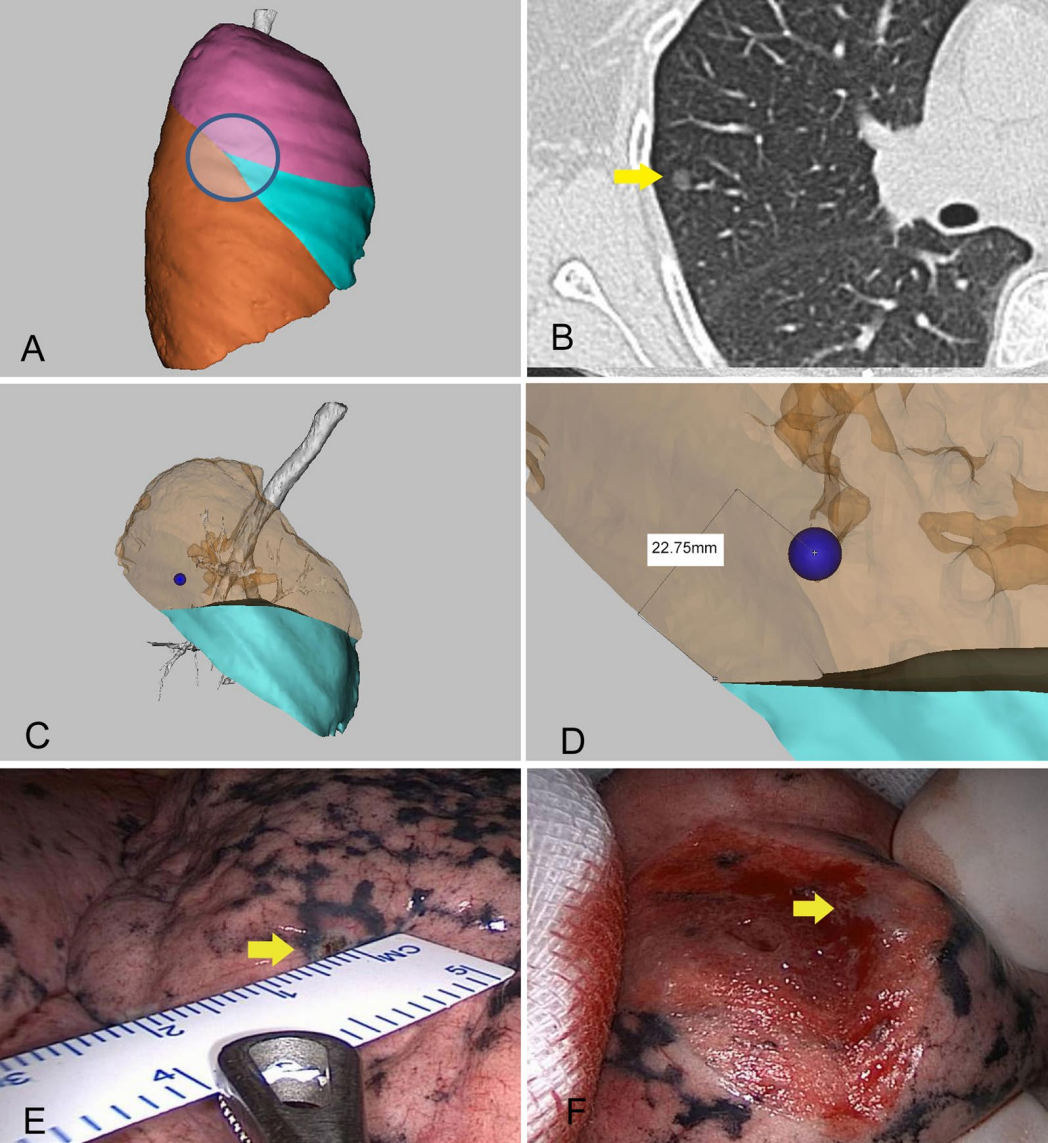
Illustration of the localisation procedure in patient 2. **A** Three-dimensional computed tomography lung reconstruction of the right lung showing the Y point and the surrounding area to be easily identified (circle). **B** A computed tomography image showing a ground-glass opacity (yellow arrow) in the right upper lobe. **C** Three-dimensional computed tomography lung reconstruction showing the nodule (yellow arrow) near the Y point. **D** Measuring the distance from the nodule to the Y point on the three-dimensional model. **E** An intraoperative view showing measurement of the distance of the nodule relative to the Y point with a sterile ruler. **F** A resected specimen showing adequate surgical margins (arrow). The pathology report revealed minimally invasive adenocarcinoma

When the lungs have not collapsed, for some fixed anatomic landmarks, such as the so-called Y point, i.e., the intersection of the oblique and horizontal fissures, a burn mark is made with the electrotome on the visceral pleura at this site. The lungs then collapse partially, and the distance of the nodule relative to the Y point is measured with a sterile ruler. The wedge resection is then performed (Fig. 3).

##### Nodules near the interlobular fissure

**Fig. 4:**
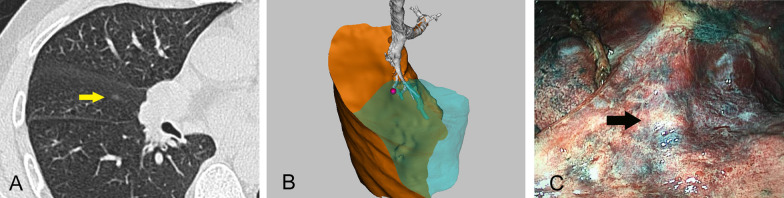
Illustration of the localisation procedure in patient 3. **A** A computed tomography image showing a ground-glass opacity (yellow arrow) in the right middle lobe. **B** Three-dimensional computed tomography lung reconstruction showing that the nodule is far from the pleura but near the horizontal fissure. **C** An intraoperative view showing that the actual position of the nodule (arrow) is consistent with the position in the three-dimensional model and can be easily identified. The pathology report revealed minimally invasive adenocarcinoma

There are difficulties and risks associated with puncture positioning under CT guidance for nodules near the interlobular fissure, especially deeper nodules. However, these nodules can be visually localised in the 3D reconstruction model (Fig. 4).

##### Nodules near the lung plane boundary or impression

**Fig. 5:**
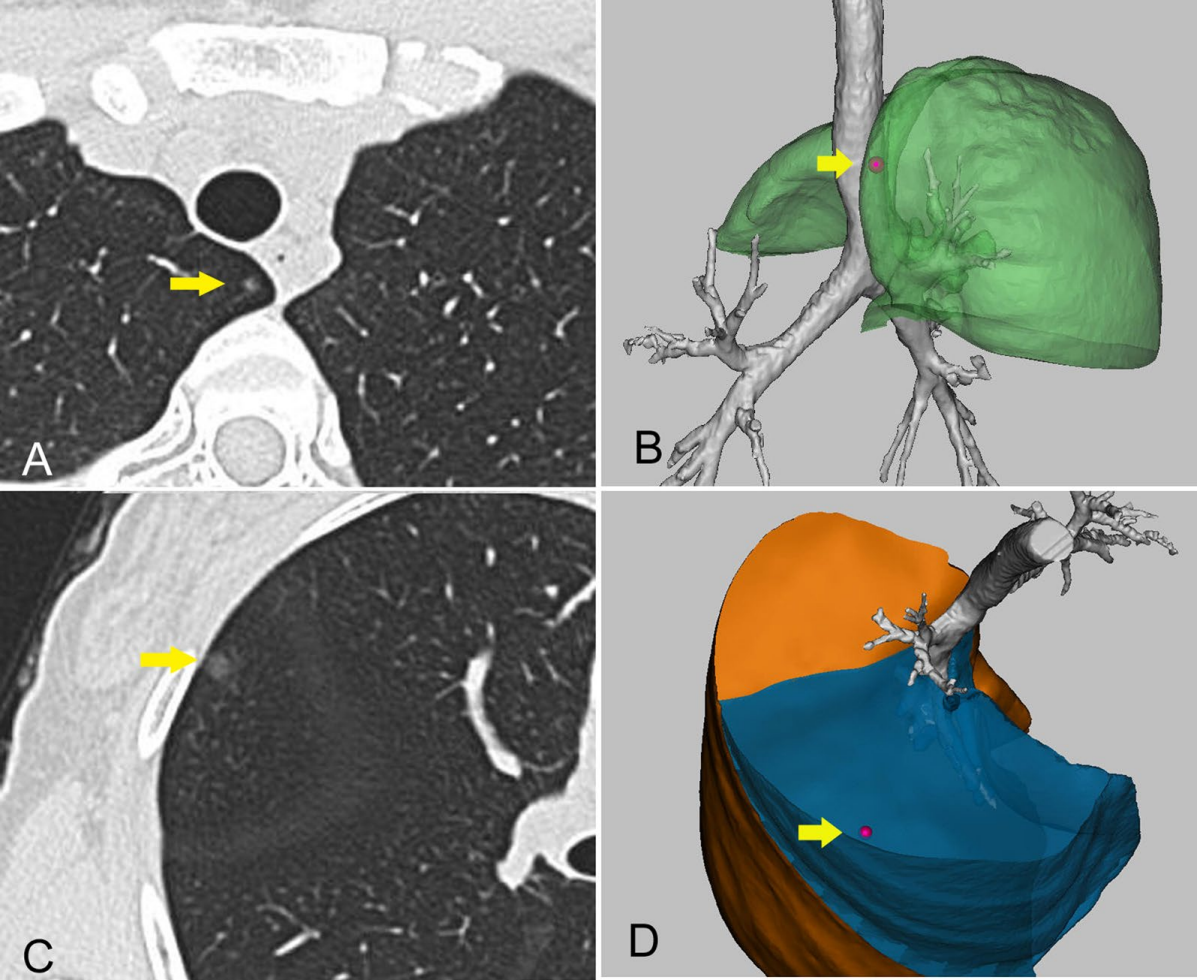
Illustration of the localisation procedure in patient 4. **A**, **C** Computed tomography images showing two nodules located in the upper lobe and middle lobe of the right lung. **B**, **D** Three-dimensional computed tomography lung reconstruction showing the nodules near the lung plane boundary, which can also be easily identified. The final histology confirmed minimally invasive adenocarcinoma (nodule A) and adenocarcinoma in situ (nodule B)

Nodules at the boundary of the lung plane are difficult to locate accurately on a two-dimensional CT image but their positions can be clearly located after 3D reconstruction (Fig. 5). Furthermore, nodules near the impression for other organs, such as the first rib, superior vena cava, heart, azygos, and oesophagus, can also be identified easily on 3D reconstruction models.

#### Deeper nodules

**Fig. 6:**
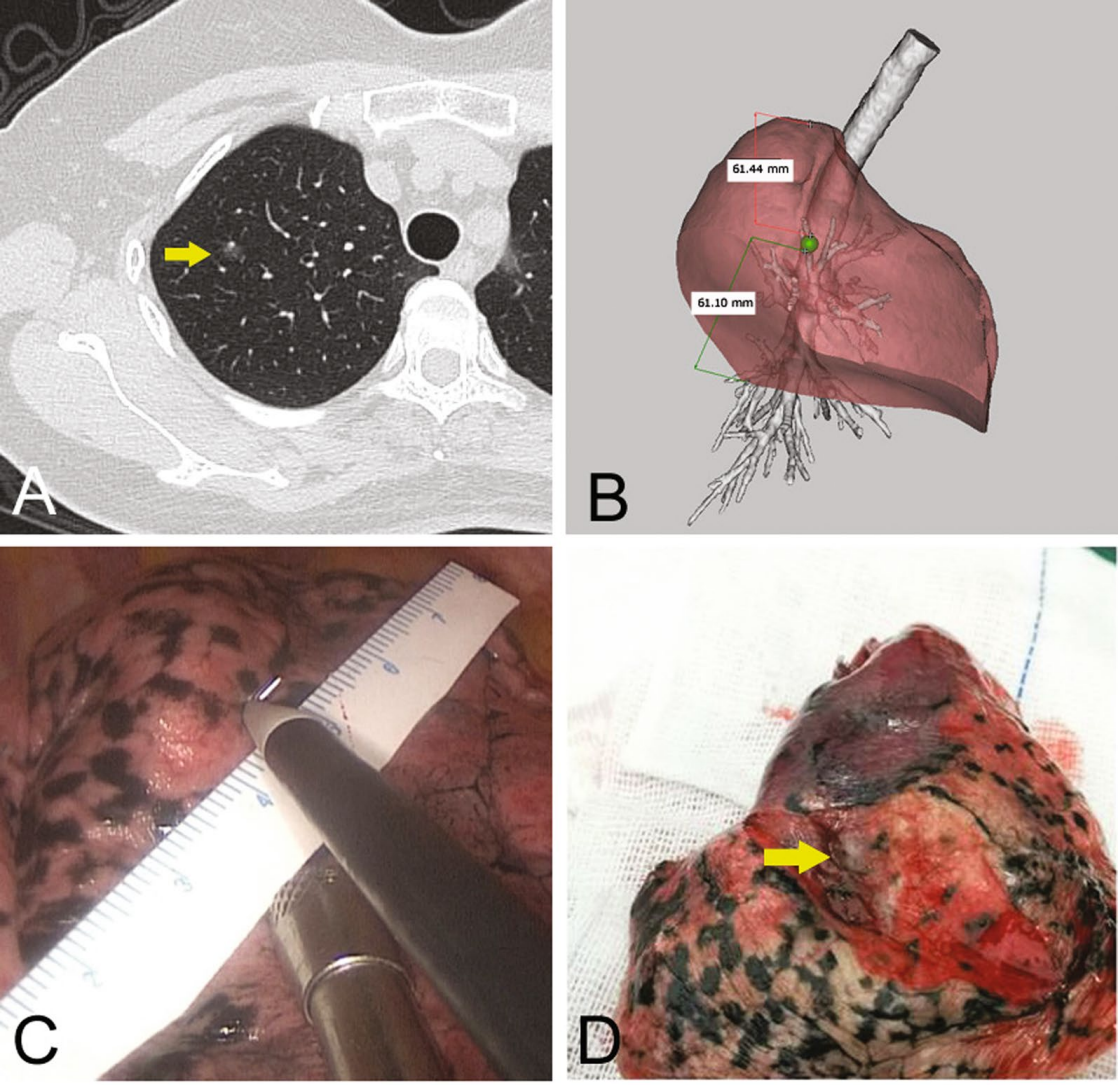
Illustration of the localisation procedure in patient 5. **A** A computed tomography image showing a nodule (yellow arrow) in the right upper lobe. **B** Three-dimensional computed tomography lung reconstruction showing that the nodule is located in the upper lobe of the right lung, far from the pleura, but that the projection position of the nodule on the lung surface is approximately the middle of the upper and lower boundaries. Three-dimensional computed tomography bronchography and angiography suggest that the nodules are located at the apical segment of the right upper lobe but close to the posterior segment. **C** Localisation is based on the distance between the nodule and the upper and lower boundaries and a burn mark made with an electrotome. **D** The resected specimen after apical segmentectomy shows adequate surgical margins (arrow). The final histology confirmed this lesion to be minimally invasive adenocarcinoma

These nodules often require lung segmentectomy, even combined segmentectomy. We can perform 3D reconstruction of the lobes, bronchial vessels, and other anatomic structures to simulate segmentectomy preoperatively. Localisation by 3D-CTLR allows identification of safe surgical margins, especially for lung nodules located in the plane between segments or subsegments, and then allows the anatomic resection of the targeted segment to be minimised while meeting oncologic requirements (Fig. 6).

### Uniportal video-assisted thoracoscopic surgery

The details of uniportal VATS were fully described in a previous study [10, 11]. Briefly, the patient is placed in the lateral decubitus position after anaesthesia. A single 3–4-cm skin incision is made in the 5th intercostal space along the anterior axillary line. Wedge resection, segmentectomy, or lobectomy is performed for the major lesion depending on its size and location. Intraoperative pathologic analysis of frozen sections is routinely performed for all patients. Thoracoscopic sublobar resections are the only option when atypical adenomatous hyperplasia (AAH), adenocarcinoma in situ (AIS), minimally invasive adenocarcinoma (MIA), or a benign nodule is reported. However, if the frozen section indicates invasive adenocarcinoma (IA), a lobectomy may be performed depending on the results of preoperative pulmonary function tests. Segmental resection is considered for nodules that are in a deep position and cannot be removed by wedge resection. Lobectomy may be considered in some patients if there are multiple nodules in the same lobe.

### Statistical analysis

The continuous data are presented as the mean ± standard deviation (range) and the categorical data as the frequency and percentage. All statistical analyses were performed using SPSS for Windows software (version 24; IBM Corp., Armonk, NY, USA). All statistical analyses were 2-tailed, and a p-value of 0.05 was considered statistically significant.

## Results

### Patient demographics and nodule characteristics

The patient demographics and the characteristics of the nodule at baseline are summarised in Table 1.

**Table 1:** Clinical and radiologic characteristics of the study population

	n (%) or mean ± SD (range)
Patients, n	44
Age (years)	55.6 ± 9.3 (3–77)
Sex	
Male	13 (29.5)
Female	31 (70.5)
Total number of nodules	121
Nodules per patient, n	2.75 ± 1.31 (2–8)
Nodules in each patient, n	
2	28 (63.6)
3	8 (18.2)
4	3 (6.8)
≥ 5	5 (11.4)
Nodule diameter, mm	7.67 ± 3.87 (2.57–22.84)
Distance between outer edge of nodule and pleura, mm	14.84 ± 14.43 (0–85.82)
Site of nodule	
Right upper lobe	47 (38.8)
Right middle lobe	16 (13.2)
Right lower lobe	25 (20.7)
Left upper lobe	26 (21.5)
Left lower lobe	7 (5.8)
Imaging characteristics	
Pure GGN	54 (44.6)
Pure-solid GGN (GGO ≥ 50%)	49 (40.5)
Pure-solid GGN (GGO < 50%)	8 (6.6)
Solid nodule	10 (8.3)

GGN, ground-glass nodule; GGO, ground-glass opacity; SD, standard deviation

### Localisation and surgical outcomes

Four nodules were not localised preoperatively because they were larger than 15 mm in diameter. The other nodules were located preoperatively by non-invasive visual inspection. According to the indications of CT-guided percutaneous localisation, 27 of the 121 lesions were in “blind areas,” 11 were near the mediastinal region, 11 were close to an interlobar fissure (3 horizontal, 8 oblique), and 5 were shadowed by the scapulae. However, the nodules in the “blind areas” were easy to identify using our localisation method.

All nodules were resected using uniportal VATS without conversion to mini-thoracotomy or thoracotomy. Table 2 shows the operative and postoperative results. Twenty-seven patients underwent an initial wedge resection, with 6 of these patients each undergoing two or more wedge resections. Twenty-five patients underwent segmentectomy, with 5 of these patients undergoing combined segmentectomy. Twenty-six patients underwent lobectomy, and the other 18 patients in whom lobectomy was precluded by limited pulmonary function or who had MIA, AIS, or carcinoid underwent segmentectomy or wedge resection after diagnosis on frozen section. A total of 24 patients had multiple nodules with ≥ 2 nodes in the same lung lobe, 7 patients received segmentectomies and 17 had pneumonectomies. Analysis of intraoperative frozen sections revealed that 39 patients had one or more nodules that were IA; in these cases, we also performed mediastinal lymph node dissection.

**Table 2:** Location and surgical outcomes

	n (%) or mean ± SD (range)
Location accuracy (%)	
Success	117 (100)
Type of operation (VATS)	
Wedge resection	33
Segmentectomy	25
Lobectomy	26
Postoperative morbidity	1 (2.27)
Pathologic diagnosis	
Benign	6 (4.96)
AAH	12 (9.92)
AIS	13 (10.74)
MIA	16 (13.22)
IAC	73 (60.33)
Other	1 (0.83)
Operating time, minutes	118 ± 24
Blood loss, mL	39.1 ± 22.78
Length of hospital stay, days	4.41 ± 1.56

AAH, atypical adenomatous hyperplasia; AIS, adenocarcinoma in situ; IAC, invasive adenocarcinoma; MIA, minimally invasive adenocarcinoma; SD, standard deviation; VATS, video-assisted thoracoscopic surgery

One patient developed postoperative pneumonia and underwent 9 days of antibiotic treatment. No severe complications occurred during or after VATS. There was no 30-day or in-hospital mortality.

### Pathologic diagnosis of nodules

Histologic diagnosis of lung carcinoma was classified as pre-invasive (including AAH and AIS), minimally invasive (MIA), or invasive (IA). The final pathology is shown in Table 2. The majority (95%) of lesions were malignant.

## Discussion

With the increasing use of low-dose CT for lung cancer screening, SMPLC is being encountered with increasing frequency in clinical practice [1]. There is an increasing role for surgical resection of pulmonary nodules in patients for both diagnostic and therapeutic purposes. However, reliable localisation and complete resection of these pulmonary GGOs during minimally invasive pulmonary resection represents a major clinical challenge [4, 12]. In this study, we evaluated 3D-CTLR as a non-invasive visual localisation procedure to improve localisation and resectability of GGOs. We have demonstrated this procedure to be safe and feasible for localisation of lung nodules before sublobar resection in patients with SMPLC. This approach has been most useful in patients with small and peripheral lesions and may ultimately serve as a safe and easy localisation method that can complement invasive localisation techniques.

For patients with SMPLC, when the main lesion is removed, we always try to remove the remaining small lesions and often attempt a tentative wedge resection. Intraoperative macroscopic observation and finger palpation are the most direct, simple, and safe intraoperative localisation methods for pulmonary nodules. However, nodules that are < 1 cm in size, those that are not solid or are partially solid, and those with a size smaller than the distance to the pleural surface are difficult or impossible to locate by palpation intraoperatively with thoracoscopy and often require an extended resection [1]. Segmentectomy or even lobectomy may be performed but may not always be necessary depending on the nature of these tiny nodules. Therefore, several preoperative localisation methods have been proposed to improve on intraoperative localisation of lung nodules [4, 9, 12–14]. Although the reported success rates have ranged from 68 to 100%, the most commonly used localisation techniques, i.e., CT-guided percutaneous localisation with a hookwire or microcoil, are invasive and associated with well-documented procedural complications, including radiation exposure, dislodgment, pneumothorax, and bleeding [5, 15]. The “blind areas” encountered during percutaneous localisation, including the region in the vicinity of the mediastinum, areas neighbouring the interlobar fissure, and scapulae-shadowed areas, are the major limitation of this localisation technique [6]. The aim of preoperative localisation is to avoid procedures that are more invasive; however, there are few reports concerning less invasive preoperative localisation. The present report describes a safe and feasible method for preoperative localisation of small nodules in patients with SMPLC.

As mentioned earlier, preoperative 3D-CTBA has been widely used in segmentectomy [10, 16–19]. Preoperative simulation on the 3D image is helpful for surgical planning, which includes identification of the target vessels, bronchus, and surgical margin, as well as any anatomic variations and the relationship between the nodule and the reconstructed vessels and bronchus [14, 16]. However, the above methods are not able to completely solve the problem of location of the nodules. In the absence of accurate localisation of superficial pulmonary nodules, wedge resection may be inaccurate. In our patients, we use 3D-CTLR to obtain the exact locations of multiple nodules, which can be identified intuitively and accurately based on the relationship between the nodule and a fixed anatomic landmark on the surface of the reconstructed lung.

Using 3D-CTLR, we have been able to simplify and facilitate the localisation procedure in 44 patients with SMPLC (117 nodules), and all nodules were resected successfully by VATS. Our experience demonstrates that non-invasive visual localisation of SMPLC by 3D-CTLR is feasible and safe. The advantages include a rapid learning curve and easy localisation of nodules that might otherwise be difficult for the surgeon to identify during VATS. Our technique encompasses the following characteristics of an ideal localisation method: a high accuracy rate, avoidance of the discomfort and morbidity associated with invasive localisation methods, the ability to be applied to the whole lung field, availability in most hospitals, and no additional radiation exposure to patients or health care professionals. Although each nodule has a different position in the lobes, this technique could be used in most patients with small, deep, and semi-solid nodules that would otherwise be difficult for the thoracic surgeon to localise with thoracoscopy.

We recognise that this study also has several limitations. First, the lungs are inflated on the preoperative CT scan, whereas the lung on the operative side is collapsed and retracted during thoracoscopic surgery. Although we located the nodules when the lungs were not completely collapsed, there was a slight discrepancy between their actual position and their measured position. Second, Some nodules lack fixed anatomic landmarks for accurate localisation. For those nodules, CT-guided percutaneous localisation methods may be needed. Finally, the sample size was small, and the study was retrospective and performed in a single centre. Therefore, the precision and safety of this technique needs to be evaluated in more patients in the future.

## Conclusions

Our study demonstrates that 3D-CTLR is a non-invasive visual localisation method that is safe and feasible for identifying small or non-solid pulmonary nodules that are deemed difficult to localise. This method enables surgeons to resect small, deeply located, GGO-predominant, synchronous, multiple, or indeterminate pulmonary nodules smoothly via sublobar resection in some suitable patients.

## Data Availability

The datasets used and/or analysed during the current study are available from the corresponding author on reasonable request.

## Abbreviations

3D-CTBA: Three-dimensional computed tomography bronchography and angiography
3D-CTLR: Three-dimensional computed tomography lung reconstruction
AAH: Atypical adenomatous hyperplasia
AIS: Adenocarcinoma in situ
CT: Computed tomography
GGN: Ground-glass nodule
GGO: Ground-glass opacity
IA: Invasive adenocarcinoma
MIA: Minimally invasive adenocarcinoma
MIMICS: Materialise Interactive Medical Image Control System
SMPLC: Synchronous multiple primary lung cancers
VATS: Video-assisted thoracoscopic surgery
